# Cell Adhesion to Plasma-Coated PVC

**DOI:** 10.1155/2014/132308

**Published:** 2014-08-26

**Authors:** Elidiane C. Rangel, Eduardo S. de Souza, Francine S. de Moraes, Eliana A. R. Duek, Carolina Lucchesi, Wido H. Schreiner, Steven F. Durrant, Nilson C. Cruz

**Affiliations:** ^1^Laboratório de Plasmas Tecnológicos, Universidade Estadual Paulista (UNESP), Avenida Três de Março 511, 18087-180 Sorocaba, SP, Brazil; ^2^Laboratório de Biomateriais, Departamento de Ciências Fisiológicas, Pontifícia Universidade Católica, Praça Dr. José Ermírio de Moraes 290, 18030-095 Sorocaba, SP, Brazil; ^3^Laboratório de Superfícies e Interfaces, Departamento de Física, Universidade Federal do Paraná, 81531-990 Curitiba, PR, Brazil

## Abstract

To produce environments suitable for cell culture, thin polymer films were deposited onto commercial PVC plates from radiofrequency acetylene-argon plasmas. The proportion of argon in the plasmas, P_Ar_, was varied from 5.3 to 65.8%. The adhesion and growth of Vero cells on the coated surfaces were examined for different incubation times. Cytotoxicity tests were performed using spectroscopic methods. Carbon, O, and N were detected in all the samples using XPS. Roughness remained almost unchanged in the samples prepared with 5.3 and 28.9% but tended to increase for the films deposited with P_Ar_ between 28.9 and 55.3%. Surface free energy increased with increasing P_Ar_, except for the sample prepared at 28.9% of Ar, which presented the least reactive surface. Cells proliferated on all the samples, including the bare PVC. Independently of the deposition condition there was no evidence of cytotoxicity, indicating the viability of such coatings for designing biocompatible devices.

## 1. Introduction

Currently, polymers are used for the fabrication of cheap products and devices of high aggregated value. Polymeric implants are good examples of the latter [1–3], in which the material has to satisfy specific requirements. To enable physiological activity of the medium, for example, materials of medical-grade purity are required. In such cases, the adjustment of the polymer properties is mandatory to allow its application.

Generally, such modifications are induced by changing the polymerization process and chemical composition or by using additives [3]. As in biological applications, however, the properties of the first monolayers determine the material's performance, surface modifications are often sufficient to produce suitable materials [4–6]. Treatments are based on removal or incorporation of chemical species and on topographic alterations. It is known, for instance, that surface carbonization alters cellular adhesion and proliferation [7]. Several widely employed techniques include pyrolysis [8], ion implantation [9, 10], and ultraviolet irradiation [11].

An alternative and attractive way of promoting such adjustments is through the deposition of a coating that presents the desired biological characteristics [12–14]. Surface properties are shifted to those of the film without losing the important bulk properties. The modification induced by this method may be extremely pronounced and is not limited to polymeric surfaces.

Recently, great attention has been devoted to the plasma deposition techniques, mainly due to their broad technological applications. Low temperature plasmas are composed of a variety of species, such as electrons, ions, free radicals, atoms, and molecules in excited states [15]. When a material is exposed to the plasma, it is subject to the action of such species, which are typically very reactive. Structural [16], compositional [17], and morphological [18] changes can be induced, the degree of modification being strictly related to the plasma process parameters. Therefore, the treatment can assume different characteristics depending on the plasma excitation conditions.

Plasma deposition and treatment technologies are widely employed since they are relatively simple, cheap, clean, and versatile. Surface treatments influencing the first few nanometers or deposition of films of a few microns thickness can be implemented.

In this work, the deposition of films was adopted to modify the surface properties of commercial poly(vinyl)chloride, PVC, pieces, aiming at the production of materials with properties suitable for cellular growth. The coatings were prepared in radiofrequency plasmas of acetylene-argon mixtures. While the proportions of the gases in the mixture were varied, the total pressure was maintained constant. The effects of this parameter on the cellular adhesion and proliferation, cytotoxicity, surface free energy, roughness, and chemical composition of the films were investigated.

## 2. Materials and Methods

**Figure 1:**
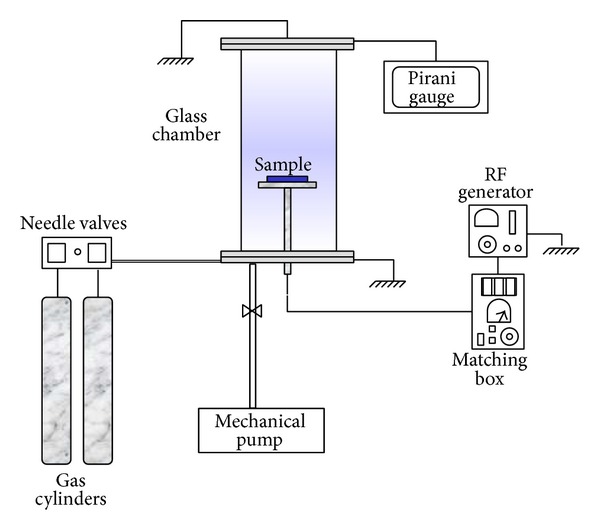
Experimental apparatus employed to deposit films onto the PVC pieces.

Thin polymer films were deposited onto commercial PVC plates in an experimental apparatus shown schematically in Figure 1.

In this system, it is possible to clean surfaces via chemical and physical ablation, to treat materials in plasmas generated from reactive gases, and to deposit films by plasma enhanced chemical vapor deposition (PECVD). The system is composed of a cylindrical glass chamber of about 2.0 L in volume. Aluminum flanges seal the top and bottom parts of the tube and possess inlets for the vacuum pump, gas lines, and pressure gauges. Plasma ignition is promoted by the application of a radiofrequency electrical signal to a stainless steel cylindrical electrode, within the chamber, which is also employed as the sample holder. The metallic flanges are normally grounded.

An 18 m^3 ^h^−1^ rotary vane pump (Edwards, E2 M-1a) is used to reduce the chamber pressure from atmospheric to about 10^−1 ^Pa. Gases are admitted via needle valves (Edwards, FCV-10k) and pressure is measured by a Pirani gauge (Edwards, APG-L). The sample holder is connected to the radiofrequency power supply (13.56 MHz) from Tokyo Hy-Power (RF-150) equipped with a matching circuit (MB-300).

Samples were prepared from commercial PVC sheets of 1.00 × 2.00 × 0.04 cm^3^. These samples were chemically cleaned in an ultrasonic bath for 1200 s using a solution of water and a detergent (DET LIMP S32). The plates were rinsed in running water and immersed in isopropyl alcohol in the ultrasonic bath. After 1200 s, they were removed and dried using a hot air blower.

Prior to each deposition, substrates were sputter-cleaned for 180 s in radiofrequency (13 Pa, 50 W) argon plasmas. Without exposing the substrates to the atmosphere, the films were deposited in argon and acetylene plasmas excited by radiofrequency power (13.56 MHz, 70 W). The concentration of argon in the gas mixture was increased from 5.3 to 65.8% while that of acetylene was proportionally decreased, yielding a total gas pressure of 2.5 Pa. Deposition times of 1800 s were used throughout.

The chemical composition of the films was studied by X-Ray Photoelectron Spectroscopy (XPS) in a VG ESCA 3000 instrument. Spectra were acquired using MgK_α_ radiation with an energy resolution of 0.8 eV. In these analyses, the C 1 s peak was taken as the reference for the energy calibration of the other species. Following background correction, high resolution spectra were deconvoluted into Gaussian curves.

Topographic profiles of the surfaces were acquired in a Veeco Dektak system. Average roughness (*R*_*a*_) was evaluated from 500 *μ*m scans taken with a 5 *μ*m tip subjected to 3.0 mg of normal force. The results presented here correspond to the average value obtained from ten different regions of the samples. Furthermore, the receptivity of the films to deionized water and methylene iodide was probed using the sessile drop technique in a Ramé-Hart 100-00 goniometer. The contact angle (*θ*) was measured in three representative regions of the samples for both compounds and the surface free energy was derived using the geometric method [19].

For the cytotoxicity studies, film was deposited on both sides of the PVC sample and placed in Petri dishes together with latex discs which were immersed in phenol. The latter were used as positive controls for cytotoxicity while the polystyrene dishes were taken as the negative ones. Incubation was performed under the same conditions described elsewhere [20]. The cytotoxicity was evaluated by the absorbance of the MTT at 570 nm measured using an Elx800-UV Bio-Tek Instruments spectrophotometer. For the analysis of cellular adhesion, the coated PVC and an as-received Teflon sample were placed in the culture dishes and kept under the same conditions employed in the cytotoxicity tests for 24 h. Teflon discs served as negative controls for cellular adhesion and polystyrene dishes as the positive controls. After incubation, using the same procedure adopted in a previous study [20], the solution was transferred to another plate and its absorbance was measured at 570 nm. To evaluate the cell-film interaction, surfaces were observed by scanning electron microscopy using a JEOL JSM-5800 LV microscope after 24, 48, and 144 h of incubation. To avoid sample charging during the experiments, surfaces were coated with a thin gold layer in a Balzers SCD 050 evaporator.

## 3. Results and Discussion

### 3.1. Surface Chemical Composition

From the full range XPS spectra of the films, it was possible to verify the presence of carbon, oxygen, and nitrogen in all the samples by the peaks at 284.6 eV (C 1 s), 532.9 eV (O 1 s), and 395.7 eV (N 1 s), respectively. Traces of titanium and chlorine were detected in the films prepared with 55.3 and 65.8% of argon in the plasma. In the spectrum of the as-received PVC, in addition to the C 1 s contribution, a peak at 203.0 eV was observed and is attributed to chlorine.

**Figure 2:**
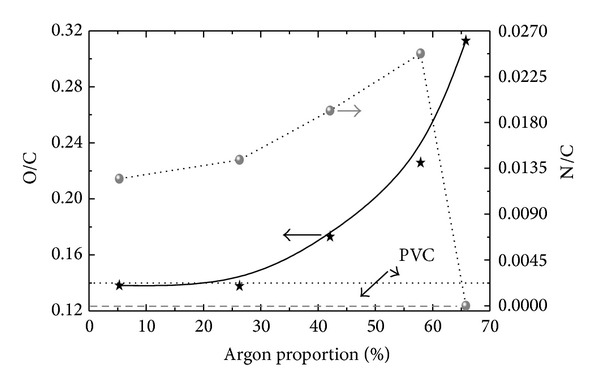
O/C and N/C atomic ratios in the samples as a function of P_Ar_. Dotted and dashed lines represent, respectively, the O/C and N/C ratios for the uncoated PVC sample.

The O/C and N/C atomic ratios were calculated from high resolution spectra of peaks due to carbon, nitrogen, and oxygen. Results are presented in Figure 2 as a function of the argon proportion in the gas feed, P_Ar_.

There is progressive enhancement in O/C with increasing P_Ar_, indicating an increase in O incorporation. For the samples prepared at the lowest argon proportions, O/C ratios are comparable to those encountered for bare PVC.

As the PVC structure does not contain nitrogen, the N/C ratio is zero in the reference sample. Nevertheless, there is progressive rise in this ratio with increasing argon proportion up to 55.3% and a sudden fall for the highest value of P_Ar_. Even though O/C and N/C initially present the same tendency, O/C ratios are about tenfold greater than those of N/C. The incorporation of O and N is ascribed in part to the residual atmosphere in the reaction chamber during film formation but mainly to postdeposition reactions with atmospheric species. In the latter case, the phenomenon is governed by the proportion of free radicals left in the structure, which, in turn, depends on a series of factors.

According to the results obtained in a parallel study [21], the proportion of C-H species in the film increases as P_Ar_ is increased from 5.3 to 28.9%. Argon introduction enhances the plasma activity and then the rate of the monomer fragmentation. For P_Ar_ above 28.9%, there is a relative shortage of monomer such that the generation of C-H groups in the plasma tends to decrease. Besides plasma reactivity and species availability, another factor that influences the concentration of C-H in the film is ion bombardment promoted by the self-bias voltage of the driven electrode. Indeed, the proportion of argon in the plasma itself influences the ion bombardment process [22]. Thus, the presence of argon affects the proportion of C-H in the solid phase [21, 22].

The bombardment of the growing layer has important implications for the overall film properties. As energetic ions cross the material, they produce, amongst other effects, bond cleavage and the liberation of volatile species [23]. In organic materials, H is preferentially lost since it constitutes lateral groups or chain termination and is weakly bonded to the structure [24]. Unstable free radicals generated in this process tend to be consumed by chain unsaturation or crosslinking or both [25]. Residual unconsumed radicals are kept active in the solid structure and react with atmospheric N and O when exposed to air. Therefore, the proportion of O- and N-containing groups in the films can be used as an indication of the radical concentration left in the film after deposition.

Since the proportion of contaminants does not change as P_Ar_ is increased from 5.3 to 28.9%, it is possible to say that crosslinking and unsaturation occur and consume the extra free radicals produced in the layer. On the other hand, the proportion of radicals and, therefore, of atmospheric contaminants grows in the films deposited with P_Ar_ above 28.9%, indicating that crosslinking and unsaturation are not prominent in these cases. Radical consumption by crosslinking and unsaturation depends on the concentration, distribution, and mobility of these species.

**Figure 3:**
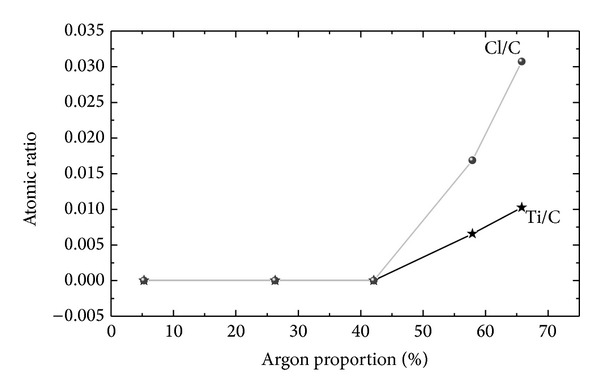
Cl/C and Ti/C atomic ratios in the films as a function of the argon proportion in the gas mixture.

The behavior of Cl/C and Ti/C atomic ratios is presented in Figure 3 as a function of P_Ar_. For films prepared with moderate argon proportions (up to 44.7%), there is no detection of Cl or Ti. For the two highest argon proportions, these elements were observed. Titanium originates from the bombardment of the glass chamber where the experiments were conducted. The increasing effectiveness of the plasma in removing species at the highest argon proportions justifies the presence of such elements in the films prepared at 55.3 and 65.8% of P_Ar_. Chlorine contamination is ascribed to migration of chlorinated species from the PVC substrate stimulated by the warming effect of the ion bombardment.

From the above results, it can be concluded that films are composed of carbon, oxygen, and nitrogen. Although XPS is not sensitive to hydrogen, this element is present in the acetylene molecule and is also a film constituent. Contamination by chlorine and titanium was observed under some conditions but only at concentrations of about a hundredth that of oxygen, the most important contaminant in these materials. Therefore, the composition of the PVC surface is altered by the deposition of the films, the degree of modification being dependent on the proportion of gases in the plasma feed.

### 3.2. Surface Energy and Roughness

**Figure 4:**
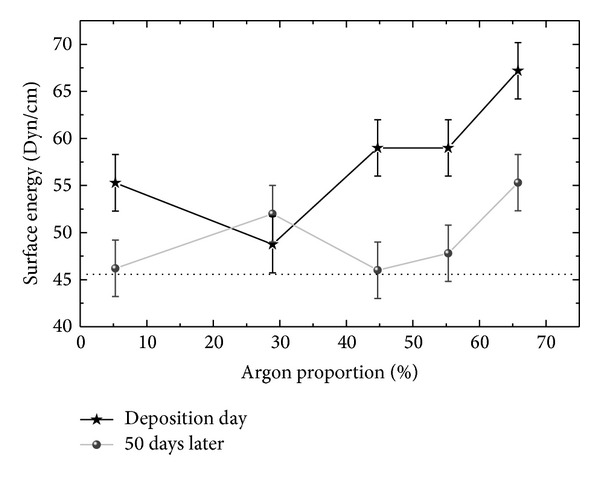
Surface free energy of the films deposited with different proportions of argon in the plasma. The dotted line represents the surface energy of the as-received PVC plate.

Figure 4 shows the surface energy, *E*_*S*_, of the samples as a function of P_Ar_, determined on the same day as the treatment and 50 days later. The *E*_*S*_ value for the reference sample is also presented in the figure by the dotted line.

Results obtained on the same day as the deposition reveal a diminution in *E*_*S*_ as P_Ar_ is increased from 5.3 to 28.9%. Subsequently, a continuous rise is observed for argon proportions above 28.9%. According to this analysis, the most receptive surface was that prepared with the greatest proportion of argon. Independently of the deposition condition, however, the coating increased the surface receptivity of the PVC to other materials.

Amongst other variables, the surface reactivity depends on the concentration of polar groups. Recombination of nitrogen and oxygen with pendant carbon bonds contributes to dipole formation. Therefore, the trends in *E*_*S*_ with P_Ar_ can be understood when the behaviors of O/C and N/C in Figure 2 are considered. As the proportion of argon increases beyond 28.9%, O/C and N/C grow, conferring a higher reactivity on the surface. It should be pointed out here that although the films deposited with 5.3 and 28.9% of Ar presented practically the same O/C values as the untreated PVC (Figure 2), their surface energies were quite different.

Through the analysis of the second curve in Figure 4, it is possible to verify that *E*_*S*_ is not stable with respect to aging time. After 50 days of contact with the atmosphere, the samples had their surface energies substantially reduced. Only for the film prepared at 28.9% of argon was the variation in *E*_*S*_ insubstantial. For all the other samples, the variations were below the detection limit (3°).

In polymers, the temporal evolution of the surface energy is very well established [25]. The phenomenon is related to movements of polar groups from the surface to deeper regions by the diffusion of species or by their rotation around the chain, reducing their effect upon compounds deposited on the surface. Such rearrangements, however, are impeded in rather rigid materials, such as highly cross-linked polymers. The connection of neighboring chains by covalent bonds promotes anchor points that limit the mobility of any species present. Therefore, the greater stability of the sample prepared at 28.9% of argon in the plasma is a consequence of the high degree of reticulation produced in this sample.

**Figure 5:**
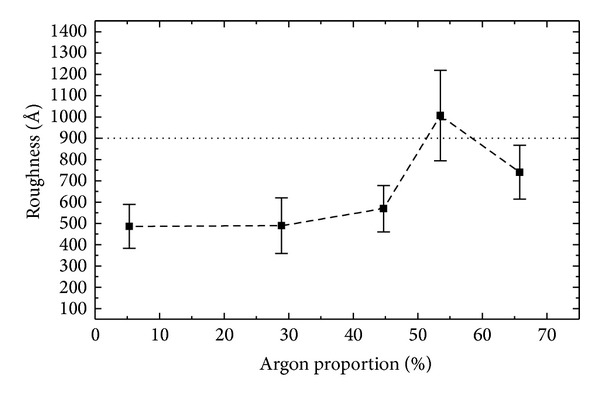
Average roughness of the films as a function of P_Ar_. The roughness value for the noncoated PVC plate is represented by the horizontal dotted line.

Aside from the effect of polar groups on *E*_*S*_, surface relief also has an effect. To investigate how this aspect influences the results presented in Figure 4, the average roughness [26], *R*_*a*_, was evaluated from the surface topographic profiles. Results, including those for the bare PVC, are depicted in Figure 5 as a function of P_Ar_.

As the argon proportion is increased from 5.3 to 28.9%, *R*_*a*_ is practically constant. A strong enhancement, however, is observed as P_Ar_ is varied from 28.9 to 55.3%. For the greatest argon proportion, there is an inversion of this tendency.

Thus, with the exception of the sample prepared with 55.3% of argon, the roughness of the PVC is decreased by film deposition. This effect can be ascribed to the intensification of the ablation process at greater proportions of Ar. The competition between the deposition and removal processes defines the growing rate and structure of the final surface. Furthermore, recoil implantation, that is, the displacement of film species to deeper regions produced by ion bombardment, also contributes to the observed effect. This interpretation does not explain the reduction of *R*_*a*_ in the last part of the curve. Indeed, further results are required to fully explain this behavior. Inspection of the plasma activity and composition, for instance, could provide important information concerning the deposition kinetics.

Comparing the surface energy and roughness results, no clear correlation between these two quantities is observed. It is interesting to note, however, the stability of *R*_*a*_ for the films prepared with 5.3 and 28.9% of Ar, while *E*_*S*_ changes significantly over the same range. This indicates the effect of chemical composition on surface wettability. The fact that the roughest surface did not present the greatest surface energy corroborates this interpretation. Variations in the chemical composition had a greater effect on the wettability than the topographic ones. The film with the most receptive surface was obtained at the highest proportion of argon in the plasma.

### 3.3. Biological Analysis

**Figure 6:**
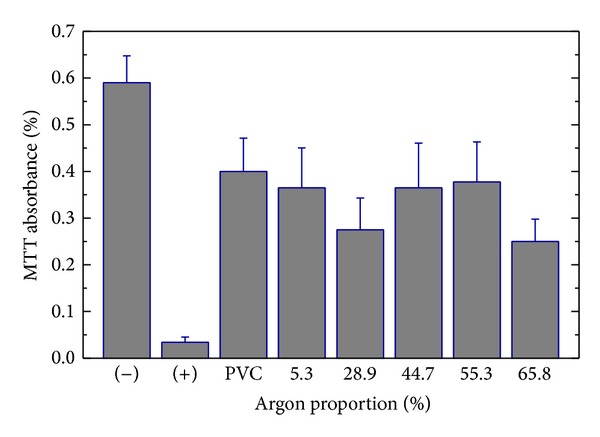
MTT absorbance as an indicator of the cytotoxicity of the bare and coated PVC samples. Films were deposited with different proportions of argon in the plasma. The absorbance of the positive and negative controls is also depicted.

The cytotoxicity results are presented in Figure 6.

The MTT absorbances for the films prepared at different P_Ar_ exhibited statistically significant differences between them; the negative control presented the highest absorbance level (*P* < 0.05) and the positive control presented the lowest. The bare PVC presented an absorbance lower than the negative control. For the coated samples, there was no significant variation in the absorbance level with respect to that of the as-received PVC. That is to say, independently of the deposition conditions, no samples present any significant level of cytotoxicity.

An important requirement for the clinical application of a material is to present good biocompatibility; that is, the material has to accomplish the necessary function without inducing undesired local or systemic effects [27]. The liberation of toxic substances at the implant can promote cellular death and thus injury. Regardless of the deposition conditions, the films employed here did not present any toxic effect to Vero cells. Even the presence of chlorine in the films deposited with the two highest proportions of argon did not affect the material's performance. Therefore, the application of the films synthesized in this work as a base for cellular growth is feasible.

**Figure 7:**
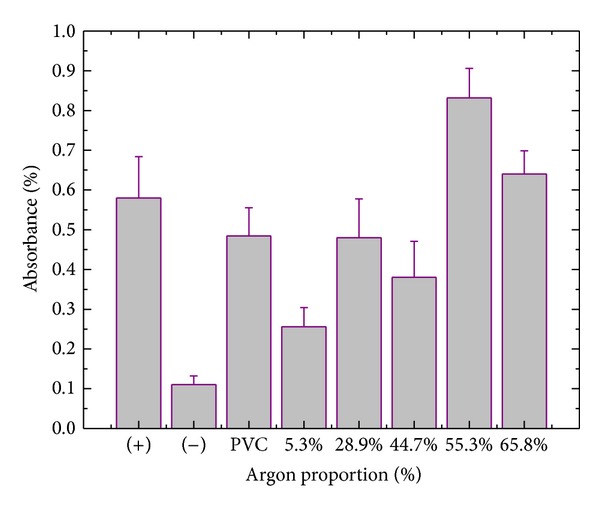
Absorbance of the MTT as an indicator of Vero cell adhesion on the bare and coated PVC samples. The films were deposited at various proportions of argon in the gas mixture. The absorbances of the positive and negative controls are also presented.

Figure 7 shows the results for the adhesion of Vero cells to the samples after 2 hours of cultivation.

The relative absorbance of the negative control is the lowest (*P* < 0.05). For the positive control, absorption levels greater than those found for the samples deposited with 5.3 and 44.7% of argon (*P* < 0.05) but lower than those detected for the films prepared with 55.3 and 65.8% of Ar (*P* < 0.05) are seen. The as-received PVC plate and the sample prepared with 28.9% of Ar revealed absorbance indices quite similar to that of the positive control. Therefore, concerning cellular adhesion, the sample which presented the worst performance was that prepared with 5.3% of argon in the plasma, whereas excellent adhesion rates were obtained for the films deposited with 55.3 and 65.8% of argon in the mixture.

The interpretation of these results lies in the interaction mechanisms of the medium and the surface. To connect cells to a surface, it is first necessary to create an extra cellular matrix of collagen, fibronectin, or any other synthetic peptides [28, 29]. The cells interact with components of the extra cellular matrix through specific cell-surface receptors [30]. It is also known that adhesion of the primary layer occurs through electrostatic forces and is then influenced by the presence of polar groups on the solid surface [31]. It is interesting to observe the correlation between the cell adhesion and the aged surface energy results. The only exception to the observed trend is the film deposited with 55.3% of argon. In this case, the interpretation relies on the greater roughness values encountered for this sample (Figure 5), producing a better environment for cellular growth than that induced in the film deposited with 65.8% of Ar.

**Figure 8:**
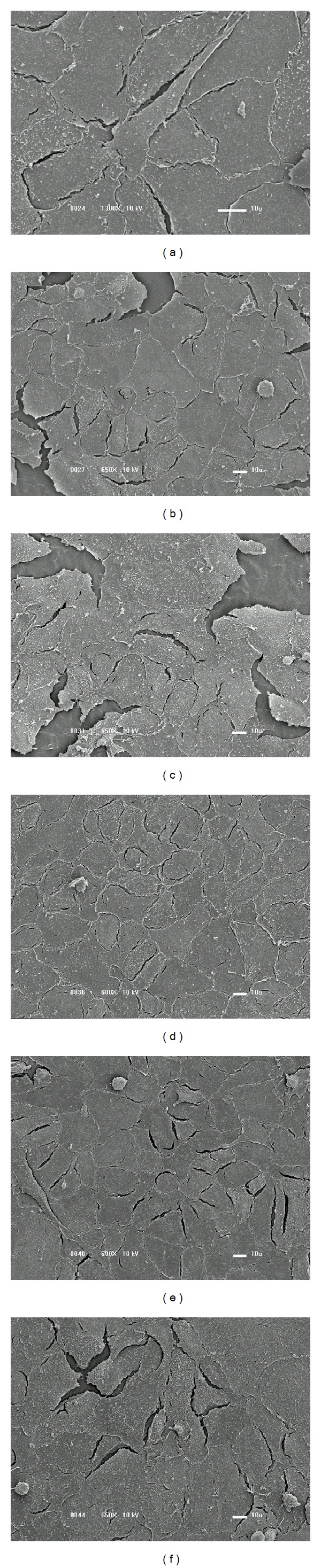
SEM surface images of the PVC samples after 24 hours of cellular cultivation. (a) Nontreated PVC plate. Sample covered with films prepared with (b) P_Ar_ = 5.3%, (c) P_Ar_ = 28.9%, (d) P_Ar_ = 44.7%, (e) P_Ar_ = 55.3%, and (f) P_Ar_ = 65.8%.

Analyses of the cell morphology and growth rate were conducted by considering the surface images. The results obtained after 24 hours of incubation are presented in Figure 8 for the as-received (a) and coated PVC samples using films deposited with 5.3% (b), 28.9% (c), 44.7% (d), 55.3% (e), and 65.8% (f) of argon in the plasma. On the bare PVC, the adhered cells practically reached the confluence after 24 hours. The cells are elongated and present cytoplasmatic projections. Vero cells which normally grow in monolayers are elongated and present particulates, being similar to fibroblastic cells [32].

In the samples deposited with 5.3 and 28.9% of Ar, cell adhesion was slightly lower, and particulates and structures similar to cell nuclei were present. As argon was employed at 44.7, 55.3, and 65.8%, the number of adhered cells increased, as shown by the greater uniformity of the cellular layers. Structures resembling cell nuclei were detected as well as high proportions of particulate material.

**Figure 9:**
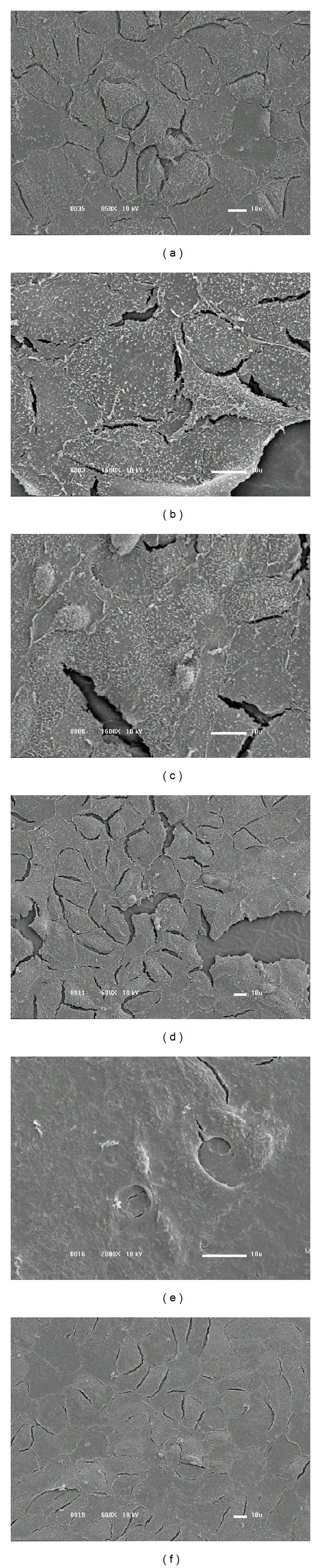
SEM surface images of the PVC samples after 48 hours of cellular cultivation. (a) Nontreated PVC plate. Sample covered with films prepared with (b) P_Ar_ = 5.3%, (c) P_Ar_ = 28.9%, (d) P_Ar_ = 44.7%, (e) P_Ar_ = 55.3%, and (f) P_Ar_ = 65.8%.

Figure 9 shows the images of the surfaces exposed for 48 hours to the cell culture. As can be observed by comparing the results of Figures 8 and 9, modifications are produced as the incubation time changes. There is a greater amount of particulate material on the surface of the films prepared with 5.3, 28.9, and 44.7% of Ar as longer incubation times are considered. On the contrary, films deposited with 55.3 and 65.8% of argon developed a layer with total confluence and practically no particulates. These results are found to be better than that of the bare PVC.

**Figure 10:**
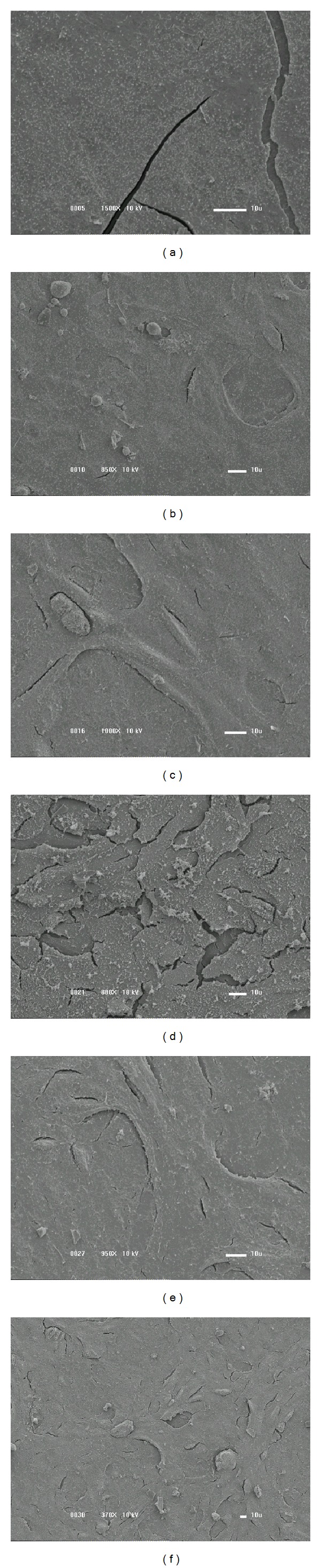
SEM surface images of PVC samples after 144 hours of cellular cultivation. (a) Nontreated PVC plate. Sample covered with films prepared with (b) P_Ar_ = 5.3%, (c) P_Ar_ = 28.9%, (d) P_Ar_ = 44.7%, (e) P_Ar_ = 55.3%, and (f) P_Ar_ = 65.8%.

Finally, the samples were inspected after 144 hours of culture. The results are presented in Figure 10. In all the samples, including the reference PVC, a monolayer of Vero cells was formed, but the morphologies depend on the deposition condition. On the bare substrate, a continuous layer with no cytoplasmatic projections was observed. The number of projections increases with increasing P_Ar_ (up to 44.7%) and then decreases for argon proportions beyond 55.3%.

The effect of roughness on the morphology of the biological coating is illustrated by the samples prepared at 44.7 and 55.3% of P_Ar_, which present practically the same surface energy but different cellular growth. Even though the surfaces can be considered chemically similar, their topographies are different, acting as a differentiation for the growth rate and morphology of the biolayer.

## 4. Conclusions

The surface of the PVC is changed by the deposition of films prepared from argon and acetylene plasmas, the degree of modification being dependent on the proportions of the gases in the mixture.

Variation of the argon proportion in the feed produces changes the ion bombardment of the growing film and thus the concentration of residual radicals. This phenomenon affects the incorporation of oxygen and nitrogen groups which, in turn, determines the surface energy of the solid. Surface microstructure and topography are also dependent on the degree of bombardment, which can be tailored via the argon proportion in the feed.

There were cellular adhesion and proliferation on all the films, independently of the preparation condition. The best performances were attained, however, with the samples prepared at the greatest argon proportions. Moreover, there was no sign of cytotoxicity, indicating that application of such films as coatings for implants is feasible.

Finally, the results encountered here demonstrate the versatility of plasma-deposited layers as biomaterials. Simple adjustments in the deposition conditions can result in materials with low or high cellular growth. This wide control is an important requirement when one considers the construction of devices for selective culture. Furthermore, as the treatment is based on the production of layers, it is unnecessary to use medical grade polymers, since the coating inhibits contact with the medium. In addition, the technique may be applied to substrates other than PVC.
